# Toxicity of Per- and Polyfluoroalkyl Substances to Aquatic Invertebrates, Planktons, and Microorganisms

**DOI:** 10.3390/ijerph192416729

**Published:** 2022-12-13

**Authors:** Tingting Ma, Chaoran Ye, Tiantian Wang, Xiuhua Li, Yongming Luo

**Affiliations:** 1 College of Resource Environment and Tourism, Hubei University of Arts and Science, Xiangyang 441053, China; 2 CAS Key Laboratory of Soil Environment and Pollution Remediation, Institute of Soil Science, Chinese Academy of Sciences, Nanjing 210008, China; 3 Hubei Key Laboratory of Low Dimensional Optoelectronic Materials and Devices, Hubei University of Arts and Science, Xiangyang 441053, China

**Keywords:** toxicity, aquatic environment, benthic organisms, *Daphnia magna*, phytoplankton

## Abstract

Per- and polyfluoroalkyl substances (PFASs), recognized worldwide as emerging pollutants, may pose a substantial threat to human health and our environment due to their stability, high concentrations, wide distribution, and easy accumulation. Ever since perfluorooctane sulfonate and perfluorooctanoic acid were recognized by the Stockholm Convention on Persistent Organic Pollutants, the public has become increasingly concerned about potential contamination and the environmental risks associated with PFASs. Ubiquitous PFAS contamination of drinking water, groundwater, surface water, and sediment has been detected, especially in areas with rapid industrial and economic development. Its accumulation in living organisms and foods has accentuated the importance of investigations into aquatic organisms at the bottom of the food chain, as the stability and integrity of the food web as well as the population quantity and structure of the aquatic ecosystem may be affected. This review provides a comprehensive summary of the toxic and toxicity-related effects of PFASs on aquatic plankton, aquatic invertebrates and microorganisms, the characteristics of different target aquatic organisms in toxicity investigations, and a feasibility evaluation of PFAS substitutes to provide valuable suggestions for further utilization and regulation of PFASs and their substitutes.

## 1. Introduction

Since their invention in the 1930s and proliferation through the 1940s and 1950s, per- and polyfluoroalkyl substances (PFASs) have been used in a variety of industries and products, including firefighting foam, aerospace technologies, furniture, cosmetics, food packaging, and paper products [1,2,3,4]. The exact number of unique PFAS compounds is unknown, with estimates ranging from four to five thousand [1]. Strong carbon–fluoride bonds render them incredibly resistant to both environmental and metabolic degradation, earning them the name of “forever chemicals” [5]. Their production peaked at 4650 t/a in 2000–2002, after which their use was discouraged due to concerns about toxic effects on humans that led to high cholesterol, thyroid disease, delayed child development and poor maternal health, ulcerative colitis, and kidney and testicular cancer [6].

Perfluorooctane sulfonate (PFOS) and perfluorooctanoic acid (PFOA) are contaminants commonly studied not only due to their long-term persistence, long-distance transportability, and common occurrence in various environmental matrices, biotas, and human populations, but also for their possible disruptive effects on the immune, metabolic and endocrine systems of living organisms [7,8,9,10]. In 2009, PFOS and related compounds were listed in the Stockholm Convention on Persistent Organic Pollutants (POPs) (Annex B), an international environmental treaty, signed in 2001 and effective from May 2004, aimed at eliminating or restricting the production and use of POPs. In 2017, PFOA was listed in Annex A, subjecting it to global regulation [8,11]. In October 2019, the fifteenth meeting of the POPs review committee, in Italy, recommended the inclusion of perfluorohexyl sulfonate (PFHxS) on the list. PFOA has been classified as a potential human carcinogen by the International Agency for Research on Cancer [12]. In light of this, short-chain analogs, like F-53B [chlorinated polyfluoroalkyl ether sulfonic acids (Cl-PFESAs)], OBS (sodium *p*-perfluorous nonenoxybenzenesulfonate), and GenX [2,3,3,3-tetrafluoro-2-(1,1,2,2,3,3,3-heptafluoropropoxy)-propanoic acid (HFPO-DA)] have attracted global attention and become widely used alternatives to PFOS and PFOA [13]. However, the prerequisite for the safe use of such substances is a thorough evaluation of the adverse ecotoxicity of commonly detected PFAS compounds and their substitutes.

In aquatic ecosystems, invertebrates, planktons, and microorganisms are essential for material circulation and energy flow. As such, the toxicity and bioaccumulation effects on aquatic organisms directly determine the survival of all other animals along this food chain, making them important study species. For example, benthic shellfish are important indicators of environmental pollution and are commonly used to study the bioavailability of various water pollutants owing to their wide distribution, large population numbers, easy capture and laboratory breeding, strong pollutant accumulation capacity, and low metabolic rate. Additionally, biological background data on these organisms are widely available [14]. Similarly, since the 1970s the zooplankton model species *Daphnia magna* has been widely used in toxicity studies due to its short life cycle, rapid reproduction rate (approximately 14 days until sexual maturity), sensitivity to toxins, and easy laboratory culturing. In addition, *D. magna* has a transparent body with visible organs that facilitates the observation of toxicity effects under an anatomical microscope. Lastly, as vital producers in the aquatic environment, microalgae are an indispensable object in the study of aquatic ecology that are often used to evaluate the health of aquatic ecosystems. The effects of pollutants on microalgae could manifest in many ways, such as effects on growth, cell morphology and structure, photosynthesis, intracellular REDOX balance, and active enzyme levels [15]. Comparisons of biomagnification of individual PFAS compounds in primary consumers (e.g., mussels) and top predators (e.g., fishes) showed that the transfer and bioaccumulation of PFASs in the aquatic ecosystem increases their toxicity, highlighting the subsequent potential health risks to humans through the consumption of PFAS-contaminated fish [16].

PFASs released from emissions can reach the sea and oceans through the air and rivers. Due to the large emission volume and high solubility of ionic PFASs, river discharge is a major concern as it is the main pathway for pollutant transmission from land to sea, including the deep sea [3]. Continuous emissions and the long persistence of PFASs have caused aquatic/marine sediments to become major collection points for PFAS pollution from multiple sources [17]. Thus, it is imperative to gain more complete and updated knowledge of the adverse effects of PFASs and their substitutes on various aquatic invertebrates, planktons, and microorganisms on different levels, such as at the genetic, cell, tissue, organ, individual, species, and colony levels. Based on the results of such investigations, reliable reference resources for the subsequent development and application of environmental remediation and management policies could be provided to governments. Moreover, accurate biological testing methods should be used, and sensitive subjects should be screened to clarify the potential toxicity of aquatic pollutants quickly, accurately, and comprehensively.

## 2. Toxicity of Per- and Polyfluoroalkyl Substances to Aquatic Invertebrates

### 2.1. Bivalvia

#### 2.1.1. Green Mussel (*Perna viridis*)

The green mussel (*P. viridis*) belongs to the phyllobranchia group of molluscs and has been widely used in environmental monitoring due to its sedentary lifestyle, filter feeding characteristics, high accumulation capacity for a variety of pollutants, and its easy identification and collection [18]. With a 96 h LC_50_ of 68.3 mg/L (Table 1), exposure to PFOS solution caused oxidative stress, including alteration of SOD activity and short-term changes in GSH, MDA, and GSH content in the coat and visceral mass of *P. viridis* [18,19]. PFOS, PFOA, PFNA, and PFDA all induced dose-dependent and bioaccumulation-correlated oxidative damage as well as DNA damage (e.g., DNA strand breaks and fragmentation, chromosomal breaks, and apoptosis), membrane instability, and reduced body weight in green mussels [20,21,22] (Table S1).

#### 2.1.2. Pearl Mussel (*Hyriopsis cumingii*)

*Hyriopsis cumingii*, a typical benthic bivalve, filter feeds on bacteria, phytoplankton, protozoa, rotifers, small cladhorns, small copepods, and organic debris. Therefore, it is more frequently exposed to PFOS than other aquatic organisms. In addition, as one of the main freshwater hyridae widely distributed across major water systems in China, its habits and structure have been studied in depth [23]. PFOS exposure caused significant oxidative compression of the gill tissue (increased SOD activity) and hepatopancreas (alteration of the activity of GSH, SOD, GST, ALT, and AST), aggravated the lipid peroxidation reaction (increased MDA content in the gill and hepatopancreas), induced the activation of apoptosis signal proteins (upregulation of caspase-3 and caspase-9), aggravated the apoptosis of cells in the tissue, and impacted the structure and function of the tissue [23] (Table 2). Further, rapid triggering of cellular detoxification and oxidative stress in the antioxidant system in the hepatopancreas of *H. cumingii* was detected during PFOS treatment; however, blockage of toxic metabolic pathways and the accumulation of oxidants may have been the origin of such cellular damage [24].

### 2.2. Other Bivalves

Understanding the effects of contaminants on freshwater mussels is critical to conservation efforts and environmental risk assessments because they are often among the species most sensitive to aquatic contaminants [25]. Concerning the two life stages of Fatmucket (*Lampsilis siliquoidea*), glochidia are 8–25 times more sensitive to PFOS exposure than juveniles, and PFOS reduced the duration of glochidia viability and probability of metamorphosis at concentrations 3000 times lower than the most sensitive acute endpoint (24 h EC_50_) [25]. PFOS caused oxidative damage to the gills of *Anodonta woodiana* by altering the activity of SOD, CAT, and POD [26]. The Asian clam, *Corbicula fluminea* (Müller, 1774), a freshwater benthic organism, has been used to evaluate the effects of toxic pollutants such as ammonia, metal(loid)s, POPs, and pharmaceuticals owing to its highly sensitive morphology, behavior, and biochemical indices, including changes in enzymatic activity and gene expression in response to chemical exposure [27]. PFOS caused a decrease in filtration rates and significant changes in the enzyme activity response of the antioxidant system (ROS promotion) in *C. fluminea* [27]. The zebra mussel (*Dreissena polymorpha*), a commonly occurring invasive species in rivers and lakes with a long life cycle (2–3 years), has been used to assess the levels of several toxicants including metals, polycyclic aromatic hydrocarbons, and polychlorinated biphenyls. It was concluded that the accumulation of these contaminants in zebra mussel tissue represents a potential hazard to organisms (i.e., fish and birds) that feed on them [28]. Although PFOS and PFOA have low bioaccumulation potential in zebra mussels [28], PFOS shows high genotoxicity, which increases with exposure concentration and time [22]. *Ruditapes philippinarum*, a beach shellfish widely occurring in coastal areas.

**Table 1:** EC_50_/LC_50_ values of target PFASs and some substitutes on aquatic invertebrates.

Invertebrate	Life Stage	Pollutant	Effect	Indicator	Value	Refs
*Perna viridis*	Adult	PFOS	LC_50_	96 h lethal	68.3 mg/L	[18,19]
		PFOS	EC_50_	Integrated genotoxicity	33 (29–37) μg/L	[22]
		PFOA			594 (341–1036) μg/L	
		PFNA			195 (144–265) μg/L	
		PFDA			78 (73–84) μg/L	
*Fatmucket*	Juvenile	PFOS	EC_50_	48 h, 96 h lethal	158.1, 158.1 mg/L	[25]
*Black sandshell*					158.1, 141.7 (80.4–249.6) mg/L	
*Fatmucket*	Glochidia	PFOS	EC_50_	24 h, 48 h lethal	16.5 (8.0–33.9), 17.7 (7.2–43.5) mg/L	
		PFOA			164.4 (116.0–232.8), 162.6 (130.6–202.3) mg/L	
*Black sandshell*		PFOS			13.5 (5.7–31.8), 17.1 (9.4–31.1) mg/L	
		PFOA			161.0 (135.8–191.0), 161.3 (135.0–192.7) mg/L	
*Perinereis nuntia*	Adult	PFOS	LC_50_	96 h lethal	64 mg/L	[29]
*Dugesia japonica*	Adult	PFOS, PFOA	LC_50_	24 h lethal	34 (30–38), 352 (331–374) mg/L	[30]
				48 h lethal	27 (24–31), 345 (325–366) mg/L	
				72 h lethal	26 (23–29, 343 (324–364) mg/L	
				96 h lethal	23 (20–25), 337 (318–357) mg/L	
*Neocaridina denticulate*		PFOS, PFOA		24 h lethal	>200, >1000 mg/L	
				48 h lethal	57 (43–75), 712 (663–764) mg/L	
				72 h lethal	20 (17–24), 546 (502–594) mg/L	
				96 h lethal	10 (9–12), 454 (418–494) mg/L	
*Physa acuta*		PFOS, PFOA		24 h lethal	271, 856 (768–954) mg/L	
				48 h lethal	233 (226–241), 732 (688–779) mg/L	
				72 h lethal	208 (197–219), 697 (661–735) mg/L	
				96 h lethal	178 (167–189), 672 (635–711) mg/L	
*Gammarus insensibilis*	Adult	PFOS	LC_50_	48 h lethal	9.99 mg/L	[31]
*Macrobrachium rosenbergii*	Adult	PFOS	LC_50_	96 h lethal	0.68 ± 0.22 mg/L	[32]
*Paracentrotus lividus*	Adult	PFOS, PFOA	LC_50_	72 h lethal	0.11, 110 mg/L	[33]
*Siriella armata*					6.9, 15.5 mg/L	
*Limnodrilus hoffmeisteri*	Adult	PFOS	EC_50_	48 h lethal pH 6.2	23.81 ± 1.14 mg/L	[34,35]
				48 h lethal pH 7.0	35.89 ± 0.49 mg/L	
				48 h lethal pH 8.0	39.80 ± 1.15 mg/L	
		PFOS	LC_50_	24 h lethal pH 5.0	45.26 mg/L	[35]
				24 h lethal pH 6.0	46.23 mg/L	
				24 h lethal pH 7.0	60.70 mg/L	
				24 h lethal pH 8.0	64.48 mg/L	
				24 h lethal pH 9.0	65.74 mg/L	

TFA, trifluoroace-tic acid; PFPrA, perfluoropropionic acid; PFBA, perfluorobutanoic acid; PFPeA perfluopentanoic acid; PFHxA, perfluorohexanoic acid.

**Table 2:** Toxicity effects of commonly detected single PFASs on aquatic invertebrates.

Classification	Damage/Effect	Impact Detail	PFOS	PFOA
Cumulative toxicity	Accumulation	Whole soft tissues	*Cf* [27] *Cv* [36] *Lh* [34,35]	
		Hepatopancreas	*Es* [37]	
		Muscle	*Es* [37]	
Developmental toxicity	Growth	Body weight	*Pv* [20]	
		Relative condition factor	*Pv* [20]	*Pv* [20]
Oxidative toxicity	Visceral mass	SOD, GSH	*Pv* [18,19]	
	Mantle	SOD, GSH	*Pv* [18,19]	
	Gill	SOD, GSH, GR, GST, POD	*Hc* [23] *Aw* [26] *Mr* [32]	
	Hepatopancreas	SOD, GSH, GR, GST	*Hc* [23,24] *Cv* [36] *Mr* [32]	
	Gastrointestinal tract	SOD, CAT	*Mr* [32]	
	Humor	SOD, CAT	*Gc* [38]	
	Whole soft tissues	CAT, EROD, GSH, GPX, ROS, POD	*Pv* [20,21] *Cf* [27] *Dj* [39] *Pn* [29] *Gi* [31] *Lh* [34,35]	*Pv* [20,21] *Rp* [40] *Dj* [39]
	Lipid peroxidation	MDA	*Pv* [18,19,21] *Hc* [23] *Cf* [27] Gi [31] *Mr* [32]	*Pv* [21]
Genetic toxicity	DNA damage	Strand break	*Pv* [20,21,22] *Pn* [29]	*Pv* [21,22] *Dj* [41]
		Point mutation	*Gc* [38]	
	Gene expression	Antioxidant enzyme	*Cf* [27] *Mj* [42]	
		Immune related (heat shock protein)	*Cf* [27] *Dj* [43]	
		P450 and phase II enzymes	*Cf* [27] *Cv* [36] *Pn* [29]	
		Neurogenesis	*Dj* [39]	*Dj* [39]
Cytotoxicity	Apoptosis	Membrane damage	*Cv* [36]	*Pv* [20]
		Caspase-3, -6 expression	*Hc* [23]	
Organ toxicity	Liver	ALT, AST	*Hc* [23,24]	
	Digestive gland	Histological alteration	*Cf* [27]	
	Gonad	Histological alteration	*Cf* [27]	
	Hepatopancreas	Histological alteration	*Es* [37]	
	Gill	Histological alteration	*Es* [37]	
Metabolic toxicity	Whole body	Oxygen consumption	*Dp* [28]	*Dp* [28]
		Multixenobiotic transporter activity	*Dp* [28]	*Dp* [28]
	Hepatopancreas	Respiration	*Es* [37]	
		Glycogen	*Es* [37]	
		CCO, LDH	*Es* [37]	
Behavioral toxicity	Predation/Feeding	Suppressed filtration rate	*Pv* [20] *Cf* [27]	
		Siphoning behavior inhibition	*Cf* [27]	
Neurology toxicity	Neurology system	Abdominal nerve cord injury		*Dj* [39]
		Cerebral ganglion	*Dj* [39]	
		Nerve of the tail	*Dj* [39]	
Immune toxicity	Critical enzyme	ACP, ALP	*Hc* [23] *Mr* [32] *Gc* [38]	
		Heat shock protein	*Cf* [27] *Dj* [43]	
Overall toxicity	Enhanced integrated biomarker response		*Pv* [22] *Cf* [27]	*Pv* [22]

*Pv, Perna viridis; Hc, Hyriopsis cumingii; Aw, Anodolffa woodiana; Cf, Corbicula fluminea; Dp, Dreissena polymorpha; Rp, Ruditapes philippinarum; Cv, Crassostrea virginica; Dj, Dugesia japonica; Pn, Perinereis nuntia; Mj, Macrophthalmus japonicus; Es, Eriocheir sinensis; Gi, Gammarus insensibilis; Mr* *, Macrobrachium rosenbergii; Gc, Glyptocidaris crenularis; Lh, Limnodrilus hoffmeisteri.*

ALT, alanine transaminase; AST, aspartate aminotransferase; ACP, acid phosphatase; ALP, alkaline phosphatase; PFCAs, perfluorinated carboxylic acids; CCO, cytochrome c oxidase; LDH, lactate dehydrogenase.is highly sensitive to pollutants and, therefore, commonly used in research that focuses on their detection [40]. PFOA exposure triggered changes of antioxidant (SOD, CAT, and POD) and biotransformation enzymes (GST, EROD, and lipid peroxide) in *R. philippinarum*. As a result, it could be considered as a potential biomarker for PFOA pollution of marine ecosystems [40]. The Eastern oyster (*Crassostrea virginica*) is a stationary filter feeding organism that can bioaccumulate contaminants in contaminated particulate matter and the surrounding water [36]. PFOS caused significant cellular lysosomal damage in oysters (*Cr. virginica*) [36] and induced histopathological alterations in the gonads and digestive glands of freshwater clams (*C. fluminea*) [27].

### 2.3. Dugesia japonica

Living in springs, streams, and lakes with good water quality, the triploderm planarian, *D. japonica*, is very sensitive to toxic substances, teratogenic factors, and environmental pollutants. Hence, it can be used as an indicator species for water quality monitoring and as a model organism for toxicological research [39]. Furthermore, research indicated that PFOA rather than PFOS induced DNA damage [41], but the expression of hsp70 mRNA could be altered by PFOS to protect against toxicity and the accumulation of adverse effects [43]. Oxidative damage and neurotoxicity were caused by PFOS/PFOA exposure, but PFOA damaged the ventral nerve cord whereas PFOS mainly affected the cranial ganglion and caudal nerve to a certain dosage; at the gene expression level, PFOS exposure inhibited the expression of otxA and otxB [39]. Both PFOS and PFOA downregulated the expression of FoxD and upregulated the expression of nlg, although this effect was delayed after PFOS exposure. Regarding the expression of FoxG, PFOA primarily affected the trunk parenchyma whereas PFOS mainly affected the brain [39].

### 2.4. Perinereis nuntia

*Polychaetes* belong to the phylum *Annelida* (*Polychaeta*) and are among the highest number of highly abundant benthic species. *Nereidae* is one of the most diverse families of polychaeta [29]. *Polychaetes* live in sediments and move slowly. Unlike the infrequent exposure that self-swimming animals or reptiles will experience, the relatively stationary mode of life of *Polychaetes* can lead to chronic exposure to various environmental toxins, and any long-term changes in benthic health can be reflected in the polychaeta community. The 96 h LC_50_ value for PFOS in *P. nuntia* was at a medium level of 64 mg/L. PFOS interfered with the CYP pathway receptor, triggered the phase I detoxification reaction system, stimulated the internal production of ROS, and indirectly caused oxidative damage, such as lipid peroxidation, which was reflected by an increase in MDA. It also induced a response in antioxidant defense systems such as SOD, CAT, GSH, GR, and GSH-Px. Phase II detoxification systems, such as GST, were also involved in PFOS metabolism, and the accumulation of oxidative damage led to severe DNA damage, causing genotoxicity [29]. At the antioxidant defense system level, SOD and GR were sensitive to PFOS stress but the effects on GSH-PX and GST were delayed; on the DNA level, PFOS induced dose-dependent short-term DNA damage, which also caused oxidative damage. Lastly, EROD activity, expression of the CYP431A1 gene in CYP2 and the CYP424A1 gene in CYP4, GST enzyme activity, and GST omega gene alternation were caused by PFOS, with CYPs and GST playing an important role in the metabolism of PFOS in *P. nuntia* [29].

### 2.5. Crustaceans

#### 2.5.1. Crabs

Crabs play a key role in material cycling and energy flow in ecosystems. As detritivores, they primarily feed on detritus that is sifted from the surface sediment [42]. The intertidal mud crab, *Macrophthalmus japonicus*, a burrowing species widely distributed across Korea, Japan, northern China, Singapore, and Australia, is one of the most abundant macrobenthic animals in many intertidal mudflats and plays an important role in purification [42]. As a typical crustacean and eurhaline organism with high economic value, *Eriocheir sinensis* is often used as a model organism for salinity studies [37]. PFOS reduced the survival rate and enhanced the oxidative metabolism of the mud crab (*M. japonicus*) [42] and caused dose- and time-dependent microstructural damage to the hepatopancreas (e.g., the lumen of the hepatopancreas tubules increases in size until the basement membrane is ruptured) and gills (e.g., thickening of gill leaf, enlargement of blood cavity, and damage to the epithelial layer) of *E. sinensis* [37]. Additionally, it has been found that the interaction between salinity levels and PFOS exposure affects the hemolymph hemocyanin content and gill respiratory metabolic enzyme cytochrome C oxidase (CCO) in *E. sinensis* [37].

#### 2.5.2. Shrimp

Besides planarians, freshwater shrimps are another pivotal detritus consumer in freshwater food webs and they play a vital role in energy and nutrient cycling in aquatic ecosystems [30]. The 96 h LC_50_ value for PFOS was 10 mg/L in green neon shrimp (*Neocaridina denticulate*) [30]. PFBA and PFBS exposure had a dose-response relationship with the enzyme activity of the antioxidant system in *N. denticulate*, and the SOD enzyme was found to be a more sensitive bioindicator than CAT and AChE [44]. In *Gammarus insensibilis*, PFOS exposure caused oxidative stress and cell damage but did not have any neurotoxic effects [31]. In *Macrobrachium rosenbergii*, the 96 h LC_50_ for PFOS stress was 0.68 ± 0.22 mg/L, resulting in oxidative damage reflected by MDA, SOD, CAT, and acid phosphatase (ACP) activity alterations. Additionally, in juvenile shrimp, 30 compounds in the gills, 19 compounds in the hepatopancreas, and 24 compounds in the gastrointestinal tract, all of which are involved in amino acid, fatty acid, and phospholipid metabolism, were adversely affected [32].

### 2.6. Echinidae

In *Paracentrotus lividus*, the acute EC_50_ values for PFOS and PFOA were 20 mg/L and 110 mg/L, respectively [33]. At the tissue level, morphological changes in *Glyptocidaris crenularis* included out stings and a dose-dependent increase of fluid inside the cell, a decrease in the number of red blood cells, short-term reduction of movement and feeding abilities, and antioxidant enzyme changes, for which SOD and ALP were sufficiently sensitive indicators. At the genetic level, low-dosage long-time exposure to PFOS increased methylation levels in *G. crenularis*, which may have subsequent genotoxic effects [38].

### 2.7. Tubifex

Exposure to Cd and PFOS caused severe damage to the antioxidant defense system of *Limnodrilus hoffmeisteri*, including effects on SOD activity, GSH levels, and MDA content alterations at pH 8.0 and pH 6.2, and the harmful effects of joint exposure were always a combination of the effects of single Cd and PFOS exposure [34]. Additionally, the pH value affected the toxicity of PFOS combinations with Cd^2+^ or Zn^2+^, especially the additive effects of bioaccumulation and oxidative stress when combined with Cd^2+^ under alkaline conditions [35].

## 3. Toxicity of Per- and Polyfluoroalkyl Substances to Plankton

### 3.1. Daphnia magna

Feeding on algae, *Daphnia magna* is a cladoceran invertebrate widely distributed across the world, and, as a primary consumer, it plays an important role in the freshwater food chain. Moreover, its short life cycle, fast reproduction rate, easy acquisition, sensitivity to toxins, and easy culture in the laboratory make it a widely used model organism for water quality benchmark studies of toxic pollutants. Thus, it has become a standard model organism for aquatic toxicology tests [45]. The toxicity of PFOS to *D. magna* is generally classified as medium, which is low for PFOA [46,47]. The 48 h immobility NOEC value for PFOS in *D. magna* was 0.8 mg/L and the 21-day lethality NOEC was 5.3 mg/L [45] (Table S2). According to the chemical toxicity grading conducted in Washington State, USA, PFOS and PFOA are classified under category D, which contains the least toxic substances. The higher toxicity of PFOS compared to PFOA was not reflected in the 48 h LC_50_ (4.23 × 10^−5^ and 2.92 × 10^−4^ mol/L) [48], 72 h LC_50_ (70.65 mg/L and 102.41 mg/L) [49], and 21 d chronic toxicity or growth inhibition in *D. magna* [48,49]. With lower aquatic toxicity, potassium perfluorobutane sulfonate (PFBSK) is a potential alternative to PFOS. The NOEC and LOEC of the 21-day reproduction test for *D. magna* were 571 mg/L and 981 mg/L, respectively [50] (Table S3). Saturated and unsaturated fluorotelomer carboxylic acids (fluorotelomer acids, FTAs) are important intermediates in the degradation of fluorotelomer alcohols into perfluorinated carboxylic acids (PFCAs). The LC_50_s for fluorotelomer unsaturated carboxylic acid (FTuCA) and saturated and unsaturated fluorotelomer carboxylic acids (FTCA) were 150 and >60 mg/L, respectively. In terms of reproduction, the EC_50_s of the time until the first brood and the mean number of offspring/females were 287 and 214 mg/L for FTuCA and 50 and 48 mg/L for FTCA, respectively [51]. The 48 h EC_50_ of C_6_HF_11_O_3_.H_3_N was >102 mg/L in *D. magna*. The NOEC for adult *D. magna* survival, the size of surviving adults, the first day of reproduction, the number of immobile neonates, and the number of live young on day 21 were >33 mg/L, >33 mg/L, >33 mg/L, 8.13 mg/L, and 4.17 mg/L, respectively [52]. The chronic NOEC for APFO after 21 d in a *D. magna* reproduction test was 12.5 mg/L, which corresponded to approximately 0.024 mg/L unionized ammonia in the test solution [53].

The dose-dependent effects of PFOS included an increase in heart rate [46,54], reduction in length [55], inhibition of the total number of offspring [46,55], and suppression of the intrinsic rate of natural growth and reproductive quality [54,55] (Table 3). The toxic effects of PFOS and PFOA on *D. magna* were time- and dose-dependent, with short-term inhibitory effects on breeding rate and increase in offspring (for PFOS, F3 is better than F1) [47,55]. However, individual fitness-related adverse effects of PFOS on *D. magna* from F1, not F0, deteriorated with an increase in the generation numbers, indicating that both continuous and discontinuous exposures can be linked to individual fitness and subsequent changes in population dynamics [56]. PFOS increased motion activity in the dark but decreased the accumulated distance, average speed, acceleration after light stimulation, and distance moved after tapping stimulation in *D. magna* [57]. There were strong synergies in the acute and chronic toxicity of PFOS and PFOA mixtures in *D. magna* [48], causing oxidative damage and inhibition of the activity of GST, CAT, and ChE enzymes [46]. Additionally, the molecular structure of some components of the antioxidant defense system contributed to these synergistic interactions [48]. The effects of the mixture on the reduced offspring rate were more evident than those of PFOS or PFOA individually [48]. PFOS was more toxic than PFNA in terms of the inhibition of growth, reproduction, and ingestion rates, as well as the activity of biomarkers, including acetylcholinesterase (AChE), SOD, and CAT, but their joint effects on growth and reproduction were antagonistic [58]. The combined exposure to PFOS and 2,2′,4,4′-tetrabromodiphenyl ether (BDE-47) had a generally antagonistic effect [46]. The toxicity effects of combined MPs [pristine and aged polystyrene (PS)] and PFC [ammonium perfluorooctanoate (APFO)] differed with the mixture ratio, with a pristine PS + APFO mixture having antagonistic effects at ratios of 4:1 and 1:4; synergistic effects at 3:1, 1:2, and 1:3, and partial additive/antagonistic effects at 2:1 and 1:1. In turn, an aged PS + APFO mixture had antagonistic effects at all ratios except for 3:1 and 1:3 ratios, at which there were partial additive/antagonistic effects [59]. The combined toxicity effects on the gut were reflected in physiological and biochemical responses such as intestinal blockage and intestinal damage under co-exposure at μg/L levels, highlighting the risks associated with long-term exposure to the pollutant mixture [59].

### 3.2. Brachionus calyciflorus

Rotifers play an important role in the dynamics of freshwater and coastal marine ecosystems and are valuable tools for assessing toxicity levels in aquatic environments [61]. In *Brachionus calyciflorus*, the acute toxicity of PFOS was approximately 2.5-fold greater than that of PFOA. PFOS was more toxic to the F0 generation than PFOA, affecting body size, juvenile period, net reproductive rate, and generation time, as well as leading to a smaller egg size, indicating transfer to offspring, reduced population density, and an increased mictic ratio [60]. PFOS affected larval stage duration, average life span, the ratio of mixed females, and inhibited egg production [62]. The 24 h LC_50_ values for trifluoroacetic acid (TFA), perfluoropropionic acid (PFPrA), PFBA, perfluoropentanoic acid (PFPeA), and perfluorohexanoic acid (PFHxA) were 70, 80, 110, 130, and 140 mg/L, respectively. The acute effects of PFCAs decreased with an increase in carbon chain length, and the mictic ratio, body size, and egg size after exposure to certain PFCAs were high [64]. PFOS affected rotifer population growth rate in a low-concentration promotion, high-concentration inhibition manner; whereas, PFOA only caused inhibition. Exposure to PFOS or PFOA increased the mictic ratios of unexposed rotifer offspring [61]. When the resting eggs of *B. calyciflorus* were incubated in media containing PFOA/PFOS, PFOS induced higher hatching rates than PFOA, since the rotifer is more sensitive to exposure during the hatching period than during the resting egg formation period [61].

### 3.3. Other Zooplankton

*Chydorus sphaericus* is a benthic cladoceran species that may behave differently from pelagic *D. magna*, but its common occurrence in the Netherlands makes it an accurate model species for studying Dutch ecosystems [65]. The 24 h EC_50_s for the seven PFCs tested, including PFBA, 5H 4:1 FTOH, PFOA, PFNA, PFUnA, and PFDoA, ranged from 0.054 to over 20 mM; whereas, the 48 h EC_50_s were lower and ranged from 0.034 mM to greater than 20 mM and the adverse effects decreased with increasing fluorinated carbon chain length (nC) [66]. The 48 h LC_50_ values for PFOA and PFOS were 78.2 mg/L and 8.8 mg/L, respectively. Twenty-one-day exposure to PFOS at concentrations as low as 0.001 mg/L led to increased mortality and reproductive defects in *D. carinata* and damaged their genetic makeup [63]. PFASs have been employed as fire suppression agents and have inhibitory effects on the reproduction and mean body length of *D. similis* at concentrations lower than those recommended by manufacturers [67]. C4 and C6 finishing agents, potential alternatives to PFOS, had fewer toxic effects on growth inhibition and the lethality of *D. pulex* [68]. The 48 h survival and immobility NOEC values for *D. pulicaria* were 46.9 and 13.6 mg/L, and the survival and immobility LC_50_ values were 169 and 134 mg/L, respectively [45].

### 3.4. Phytoplankton

#### 3.4.1. *Scenedesmus obliquus*

*Scenedesmus obliquus*, a freshwater green alga commonly used for ecotoxicology investigations, is easy to artificially culture as an indicator organism for water quality assessments [69]. The 96 h EC_50_ for PFOA in *S. obliquus* was 139.23 mg/L [69] (Table S4). The accumulation of F53-B was higher than that of PFOS and inhibited growth (72 h EC_50_ of 40.3 mg/L); therefore, this potential risk to other animals higher up the food chain deserves attention [70]. PFOS, PFDoA, and PFTeA inhibited *S. obliquus* growth rate in a dose-dependent fashion [71,72]. PFOA affected photosynthesis by decreasing chlorophyll content [69]; whereas, F-53B interfered with the normal photosynthetic process by increasing the total chlorophyll content [70] (Table 4). Single exposure to PFOS concentrations below 40 mg/L had no inhibitory effects on *S. obliquus* growth; however, long-term exposure to PFOS caused growth inhibition and the uptake of pentachlorophenol into cells but suppressed the uptake of atrazine and diuron [73].

PFOS and PFOA caused oxidative stress, including alterations in the activity of SOD, CAT, and MDA [69,72], and F-53B damaged the balance between cell oxidants and antioxidants by increasing ROS and MDA content [70]. PFOS, PFOA, PFDoA, PFTeA, and F-53B enhanced the mitochondrial membrane potential and cell membrane permeability in *S. obliquus*, and the toxicity of compounds belonging to the same class increased with an increase in carbon chain length [70,71].

#### 3.4.2. *Pseudokirchneriella subcapitata*

*Pseudokirchneriella subcapitata* is a photoautotrophic microalga that is more sensitive to some chemical agents than crustaceans and fish [53]. According to the EC_50_ values, the order of multigenerational toxicity effects on the growth of the green algae *P. subcapitata* was PFOS (35.0 mg/L), PF-656 (43.0 mg/L), PFOA (96.2 mg/L), and PFBS (20,250 mg/L) [80]. No statistically significant growth inhibition was evident from the 72 h EC_50_, and NOEC values of C_6_HF_11_O_3_.H_3_N for all endpoints were >107 mg/L [52]. The EC_50_ values for PFBA, 5H 4:1 FTOH, PFOA, PFNA, and PFDA in *P. subcapitata* photosynthesis were 1.225, 4.853, 1.807, 1.038, and 0.851 mM, respectively [77]. The 96 h inhibition of *P. subcapitata* biomass and growth rate resulting from APFO exposure was 43.24% and 14.79 [53]. The combined inhibitory toxicological effects of PFOS and triclosan on the growth rate of the green alga *P. subcapitata* were antagonistic [81].

#### 3.4.3. *Chlorella vulgaris*

Chlorella (*C. vulgaris*), a spherical single-celled freshwater alga, was one of the first life forms on earth, and is an efficient photosynthetic organism distributed across shallow waters globally. *Chlorella vulgaris* can be consumed by humans and is commonly used in fish feed [84]. The growth NOEC value for PFOS was 8.2 mg/L [45]. With 48 h, 72 h, and 96 h EC_50_ concentrations of 0.077%, 0.069%, and 0.057%, OBS fluorine protein foam extinguishing agent is severely toxic to *C. vulgaris* [84]. The 72 h EC_50_ values of PFHxA, PFHpA, PFOA, and PFNA were 12.84 ± 0.64, 5.21 ± 0.26, 2.36 ± 0.12, and 1.07 ± 0.05 mM, respectively [82]. PFOS mg/L exposure led to a concentration-dependent ROS increase, an MDA content increase, changes in chlorophyll and the structure of chloroplasts, and oxidative damage, including an increase in SOD and CAT activity at low concentrations and a decrease in their activity at higher concentrations [74].

#### 3.4.4. *Chlorella pyrenoidosa*

The 96 h EC_50_ value for PFOS in *Chlorella pyrenoidosa* was 320 mg/L. PFOS exposure resulted in a decrease in chlorophyll-a content, dissolvable protein content, and catalase activity, where the chlorophyll-a content was most affected by PFOS exposure [75]. The 96 h EC_50_ value for PFOA in *C. pyrenoidosa* was 207.46 mg/L, and PFOA induced excessive generation of ROS, triggered the activity of SOD and CAT resulting in oxidative damage, and caused permeability of the cell [78].

#### 3.4.5. *Prorocentrum lima*

Compared to other similar marine single-celled microalgae, dinoflagellates have a greater tolerance to pollutants, but low concentrations of pollutants might stimulate their growth and cause red tides [85]. F-53B, GenX, and perfluoroethylcyclohexane sulfonate (PFECHS), three substitutes for traditional PFASs, affected the growth and antioxidant systems of *Prorocentrum lima* in a low-concentration promotion, high-concentration inhibition pattern in the order of HFPO-DA > 6:2 Cl-PFESA > PFECHS [85].

#### 3.4.6. *Selenastrum capricornutum*

The autotroph inhibition of growth NOEC value for PFOS was 5.3 mg/L in *Selenastrum capricornutum*, a model organism commonly used in ecotoxicological research [45]. The 96 h EC_50_ value for PFOA was 190.99 mg/L, PFOA exposure decreased the permeability of cell membranes and the chlorophyll concentration mirrored the trends of algal growth along with stimulation of SOD and CAT activity [78].

#### 3.4.7. Other Algae

The diversity of different microalga as primary producers ensures the stability of the aquatic food web. In microalga (*Isochrysis galbana*), acute EC_50_ values for PFOS and PFOA were 37.5 mg/L and 163.6 mg/L [33]. EC_50_ values for PFHxA, PFHpA, PFOA, and PFNA in the diatom *Skeletonema marinoi* and the blue-green alga *Geitlerinema amphibium* were 4.72 ± 0.23, 2.40 ± 0.12, 0.89 ± 0.04, and 0.42 ± 0.02 mM, and 3.18 ± 0.16, 1.42 ± 0.07, 0.60 ± 0.03, and 0.28 ± 0.01 mM, respectively [82]. With the additive interaction of two target pollutants, the 96 h EC_50_ values in the algae *Dunaliella salina* and *Phaeodactylum tricornutum* were 668.671 mg/L and 351.775 mg/L for PFOA, and 156.585 mg/L and 65.127 mg/L for PFNA, indicating that PFNA had a greater inhibitory effect on growth and that *P. tricornutum* was more sensitive than *Dunaliella salina* [83]. *Myriophyllum sibiricum* was more sensitive to exposure to single PFOS and PFOA than *Myriophyllum spicatum*, as reflected by the EC_50_ values of plant length, root number, root length, wet mass, dry mass, photosynthetic efficiency, and those of the related pigments chlorophyll-a, chlorophyll-b, and carotenoids [76,79]. Both PFOS and PF-656 were non-toxic to *Anabaena* CPB4337 [80]; based on the EC_50_ bioluminescence test, the combined toxicity of a mixture of equal parts PFOS and PFOA had an antagonistic effect [86]. C4 and C6 finishing agents had small toxic inhibitory effects on growth and lethality in *Scenedesmus quadricanda* [68].

#### 3.4.8. Protozoa

As the lowest single-celled organisms, protozoa have short life cycles, ranging from less than a day to up to five days, which makes them good model organisms for ecotoxicity studies. PFOS prolonged backward swimming more than PFOA did, with chain length, critical micelle concentration, and electric charge being major determinants of backward swimming duration in *Paramecium caudatum* [87].

#### 3.4.9. Lemna

Representing aquatic producers, *Lemna* form dense floating communities on the water surface and usually dominate the local community. The growth NOEC value for PFOS was 6.6 mg/L in the floating macrophyte *Lemna gibba* [45]. C4 and C6 finishing agents had weak toxic effects on growth rate and lethality in *Soirodela polyrhiza*; therefore, they have been suggested as potential alternatives to PFOS [68].

## 4. Toxicity of Per- and Polyfluoroalkyl Substances to Microorganisms

### 4.1. Luminescent Bacteria

Bioluminescence in the marine bacterium *Vibrio fischeri*, a classic ecotoxicity study organism, was inhibited by a homologous series of perfluorinated carboxylic acids: PFHxA, PFHpA, PFOA, PFNA, and PFDA [88]. Both PFOS and PF-656 were not toxic, with a 12% luminescence inhibition at 500 mg/L and a 15% luminescence inhibition at 250 mg/L, respectively. However, after a 15 min luminescence inhibition ecotoxicity test, the EC_50_ values for PFOA and PFBS were 524 mg/L and 17,520 mg/L, respectively [80]. At concentrations below 7.133 × 10^−3^ mol/L, PFOA inhibited luminous intensity in freshwater luminescent bacteria (*Vibrio qinghaiensis* sp.-Q67), and PFOS stimulated luminous intensity [89].

### 4.2. Other Bacteria

PFASs can be incorporated into the phospholipid bilayer membrane of bacteria and alter the semi-permeable membrane. The permeability of a semi-membrane-permeable dye has been shown to be dose- and fluorinated-carbon-number-dependent [90,91]. In contrast to microplastic exposure alone, co-exposure to perfluorooctane sulfonate microplastics and PFOS reduced the oxidative stress and cell permeability of bacteria [92].

## 5. Discussion

### 5.1. Comparison of PFAS Toxicity and Related Factors

Eight-carbon backbone PFOS and PFOA are two of the most widely used PFASs, and their toxicity to aquatic environments has been the focus of many studies [86,93]. Due to their persistence in the environment, bioaccumulation within the food chain, and potential toxicity to the aquatic environment [93], PFOSs have been a long-standing focus of attention for international organizations and are the most frequently investigated and most commonly detected PFASs. In general, PFOS is categorized as being moderately-acutely toxic and slightly chronically toxic to aquatic organisms; whereas, PFOA is categorized as both acutely, chronically, and practically nontoxic [94,95]. PFOS showed higher genotoxicity in zebra mussels (*D. polymorpha*) than PFOA, although both bioaccumulation levels were relatively low [21,28], and higher acute toxicity in rotifer *B. calyciflorus* [60,61] and *P. lividus* [33] populations, although PFOA but not PFOS could induce DNA damage in planarians *D. japonica* [41]. Additionally, their toxic effects on the nervous system have been shown to vary with location [39]. However, there are special situations in which the toxicity of PFOA exceeds that of PFOS. For example, the 96 h EC_50_ for PFOA exposure in *Chlorella pyrenoidosa* was 1/3 lower than that of PFOS, and PFOS was not toxic to *V. fischeri* even though the luminescence inhibition EC_50_ value for PFOA was 524 mg/L [80].

Notably, the toxic effects of the target PFASs are dependent on several factors. For example, PFOS caused DNA damage in the coelomocyte *P. nuntia* [29] and changed out sting morphology and stimulated fluid influx into the cells of *Glyptocidaris crenularis* [38]. Further, PFOS accelerated the heart rate [46,54], reduced length [55], decreased total offspring numbers [46,55], and suppressed intrinsic rates of natural growth in *D. magna* [54,55]. An inhibition of growth rate in *S. obliquus* (also in PFDoA and PFTeA exposure) [71,72], as well as an increase in ROS and MDA content, destruction of chlorophyll and chloroplast structure, and induction of oxidative damage in *C. vulgaris* [74] have also been found on exposure to PFOS. Considering the two correlation factors, PFOS caused dose- and time-dependent microstructural damage to the hepatopancreas and gills in *E. sinensis* [37]. Further, PFOS or PFOA exposure caused time- and dose-dependent inhibition of breeding and intrinsic growth rates in *D. magna* offspring [47,55]. PFOS, PFOA, PFNA, and PFDA all induced dose-dependent bioaccumulation-correlated oxidative damage, DNA damage, membrane disturbance, and body weight inhibition in green mussels (*P. viridis*) [20,21,22], and fluorinated carbon affected the membrane permeability in bacteria [90,91].

Furthermore, nonlinear changes in toxicity and pollutant concentrations are possible. For example, PFOS led to low-concentration promotion and high-concentration inhibition of SOD and CAT activity in *C. vulgaris* [74], and inhibited the *B. calyciflorus* population growth rate, which was the only inhibitory effect of PFOA [61]. HFPO-DA, 6:2 Cl-PFESA, and PFECHS had low-concentration promotion and high-concentration inhibition effects on the growth and antioxidant systems of *P. lima* [85]. Notably, the presence of PFOS in combination with other pollutants can have significant effects. PFOS influenced the cellular uptake and toxicity levels of structurally different compounds in dissimilar manners and may have increased the accessibility and toxicity of more hydrophobic compounds to cells [73]. The toxic effect of PFOS also manifested in combination with other pollutants regardless of the degree of toxicity of other individual pollutants, and the presence of PFOS changed their apparent toxicity [15].

Additionally, PFAS accumulation seems to be tissue-specific and more commonly detected in protein-rich tissues, such as the blood, liver, muscle, and gonads [17]. PFAS with longer chain lengths (e.g., C8) tend to be more toxic than those with shorter chain lengths (e.g., C4) [77]. The presence of high-energy carbon-fluorine bonds in perfluoro compounds increases their environmental persistence and there is a distinct relationship between hydrophobicity and toxicity. For example, log EC_50_ values were highly linearly correlated with both the number of carbon atoms in the perfluoroalkyl chain and the partition coefficients [82]. Owing to the increased hydrophobicity resulting in a higher bioaccumulation potential for chemicals with longer carbon chains and the differences in functional groups, PFCs with a sulfonate group have a larger toxic potential than those with a carboxyl group. It has been suggested that for every extra perfluoromethylene group in the alkyl chain, the toxicity increases two-fold [82]. PFAS with an average BCF of approximately 4000 (PFOS), 200 (PFHxS), 50 (PFOA), and 0.8 (PFBA) L/kg dry weight suggests that both the alkyl chain length and the functional group influence the toxicokinetics (TK). Additionally, PFASs can affect the toxicity of other compounds based on their lipophilicity [96]. For example, PFASs have been shown to penetrate the phospholipid bilayer membrane and enhance permeability, which facilitated the penetration of other pollutants into the small intestine epithelium and the circulatory system [17]. However, the situation is different regarding PFOSs, because PFOS can promote extracellular polymer production, consequently reducing the toxic entry of microplastics into bacteria [92]. Even though long-chain PFCs are highly bio-accumulative in aquatic environments [97], research on the environmental accumulation and adverse effects of alternative PFCs has received increasing attention.

Although the toxicity degree and main effects of each PFAS compound differ to some extent, for advanced consumers in aquatic ecosystems the general mechanism of toxicity of PFASs after entering organisms is as follows. First, they induce a series of stress reactions in cells, including the response of various antioxidant enzymes of phase I detoxification systems, thus producing excess ROS [98]. Subsequently, induced by metabolites, phase II detoxification systems, such as GST, also participate in the metabolism of PFASs and with the accumulation of oxidative stress, DNA is severely damaged and genetic toxicity ensues. In comparison, interference with photosynthesis such as the destruction of chloroplasts and the reduction of chlorophyll-a, -b and carotenoid contents, in producers can have serious effects on their survival rate. Additionally, variations in water salinity and pH conditions have been shown to have a significant impact on the toxic effects of PFAS pollutants; therefore, it is necessary to conduct toxicity risk assessments tailored to the water conditions prevalent at the study site [37].

Notably, the data on LC_50_ and EC_50_ in different target organisms often comes from experiments conducted using non-environmental concentrations so that the relationship between the environmental and experimental concentrations of PFASs can be used as an indicator of the relevance of the study. Overall, since PFOS is generally more toxic than PFOA, the experimental LC_50_ and EC_50_ values for PFOA are often even higher than those for PFOS. As a result, the data on PFOS might be more suitable for investigations of theoretical and actual values. Taking PFOS as an example, we know that its concentration in surface waters across the United States is between <LOD and 8.97 mg/L [99]. In Table 1, the LC_50_ for PFOS in different invertebrates ranges from 0.11 mg/L to 271 mg/L, with most values exceeding 10 mg/L; whereas, the EC_50_ value for genetic toxicity was 33 µg/L, which is within the range of the actual concentrations found across the United States. In the Asia-Pacific Region, the maximum concentrations of single PFOS and PFOA in water and wastewater were below 0.06 mg/L [100], which is much lower than the levels found across the United States. In contrast, the LC_50_ values reported for PFOS effects on zooplankton, presented in Table S2, were between 8.8 mg/L and 150.34 mg/L, and the overall EC_50_ value was 28.65 mg/L. These results indicate that acute toxicity tests of LC_50_ and EC_50_ should be replaced by toxicological studies, including investigations of cyto- and genotoxicity, that focus on sub-optimal chronic exposures closer to the actual pollution levels of target PFASs.

### 5.2. Differences between Experimental Organisms

The sensitivity of an experimental organism is crucial for achieving reliable experimental results. Regarding invertebrates, green neon shrimp (*N. denticulate*) were more sensitive to PFOS (96 h LC_50_ of 10 mg/L) than aquatic snails (*Physa acuta*), freshwater planarians (*D. japonica*), and water fleas (*D. magna*), and the aquatic snail (*Physa acuta*) showed higher resistance to PFOS and PFOA toxicity over each exposure period than the other three freshwater organisms [30]. *Moina macrocopa* exhibited greater sensitivity than *D. magna* to both PFOS and PFOA in both acute and chronic exposures [101]. Freshwater algae, *C. vulgaris*, have been found to be highly sensitive to 6:2, 8:2, and 10:2 saturated fluorotelomer carboxylic acids (FTCAs) and unsaturated (FTuCA) fluorotelomer carboxylic acids (FTuCA) [102]. Blue-green algae and diatoms were far more sensitive to PFCAs than green algae, likely due to differences in cell wall structure [82]. With a 50% inhibition of growth (IC_50_) value of 31.1 mg/L, *Lemna gibba* is much more sensitive to PFOS than the green algae *S. capricornutum*, *C. vulgaris*, *D. magna*, and *D. pulicaria* [45]. Although fish could act as a good model due to their genetic similarity with humans, the investigation of toxicity to plankton is a better key component for investigations of changes in species communities, material cycling, and energy flow in aquatic ecosystems. In particular, the toxicity and absorption characteristics of aquatic organisms, such as phytoplankton and floating plants, could be an important basis for subsequent studies on bioremediation of aquatic pollution caused by PFASs. For example, living diatoms (*Chaetoceros muelleri*) have been employed as biological support for nanoscale zero-valent iron to degrade PFOA and PFOS [103]. The hard-to-replace advantage is that organisms such as living diatoms can use the carbon dioxide released during PFAS degradation for photosynthesis under water. Living diatoms have a naturally high affinity for zero-valent iron and generate ROS under stressful conditions, which is beneficial for PFAS degradation [104]. Bioaccumulated PFASs in aquatic environments can be transferred to plants via diffusion into their root systems [105]. This indicates that the sediments could be storing various types of aquatic pollutants at high concentrations, and it is imperative to carry out further studies of the bioaccumulation and toxicity effects of PFASs on submerged and emergent plants to improve the environmental health remediation of aquatic environments.

### 5.3. Application of Substitutes

In terms of the overall research frequency, PFOS and PFOA appear in most (Table 1, Table 2, Table 3 and Table 4 and Tables S1–S4) of the total toxicity investigations, which not only indicates serious pollution but also highlights the significance of screening substitutes for them. F-53B and OBS are the two of the most frequently used substitutes for PFOS, followed by substitutes such as perfluorinated butyl organic ammonium salt cationic surfactants, textile finishing agents, C4 finishing agents, C6 finishing agents [68], and PFBSK [50]. PFBA, PFBS, FC-98 (PFECHS), GenX (HFPO-DA), and F-53B, among others, have been used as alternatives to PFOS and PFOA [13].

The order of accumulation was found to be F-53B > PFOS > OBS [70], and the orders of toxicity were GenX > F-53B > FC-98 [13], PFOS > PFBSK [50], and textile finishing agent > perfluorinated butyl organic ammonium salt surfactant > PFOS > C4 finishing agent > C6 finishing agent [68]. Most toxicity experiments utilize no more than four test organisms, thus limiting the potential to define the exact toxicity sequence of PFAS substitutes. For example, in an investigation on the effects of perfluorinated butyl organic ammonium salt cationic surfactants on the target organisms *Soirodela polyrhiza*, *Scenedesmus brananda*, and *D. pulex*, almost no toxicity was observed [68]. Therefore, to ensure the reliability of any conclusions, investigations of toxic effects on aquatic organisms should be systematically designed and carried out at multiple trophic levels to confirm that the classification of toxicity levels is not determined by the experimental results from only a single model organism. Nevertheless, based on the research conducted to date, we can conclude that the application of F-53B, GenX, FC-98, textile finishing agent, and perfluorinated butyl organic ammonium salt surfactant as alternatives is still questionable.

## 6. Conclusions

Alkyl chain length and functional groups influence the toxicity of different PFASs and their bioaccumulation ability is positively correlated with their chain length. Lethal effects, growth inhibition, and other toxic effects can pose as sensitive indicators of PFAS toxicity to different aquatic organisms such as invertebrates, plankton, and microorganisms. Although the application of substitutes including OBS, PFBSK, C4 finishing agent, and C6 finishing agent could be relatively harmless, their safety should be confirmed by further toxicity tests on sensitive organisms of different types and trophic levels. While continuing to strengthen the management and control of the use of PFASs and their substitutes, the remediation of pollution of aquatic environments should also be given importance, specifically by focusing on the remediation of sediments as the key process in reducing long-term accumulation and potential environmental risk. Bioaccumulation and toxicity tests on submerged and emergent plants could be a promising starting point to solve the PFASs contamination problem in the future and could potentially provide an environmentally sustainable solution.

## Figures and Tables

**Table 3:** Toxicity effects of commonly detected single PFASs on zooplankton.

Classification	Damage/Effect	Impact Detail	PFOS	PFOA
Developmental toxicity	Growth	Body weight	*Dm* [56]	
		Body size/length	*Dm* [48,55,58]	*Dm* [48] *Bc* [60]
Reproductive toxicity	Population	Survival rate	*Dm* [45,46,55,58]	
		Growth rate abnormal	*Bc* [61]	*Bc* [61]
		Life history variability	*Bc* [60,62]	
		Average lifetime	*Bc* [62]	
		Total number per brood	*Dm* [45,46,48,55,58]	*Dm* [48]
		Time of first brood	*Dm* [45,46,48,55,56,58]	*Dm* [48]
		Number of first brood	*Dm* [45,46,55,58]	
		Innate rate	*Dm* [55,56]	
		Density	*Bc* [60]	*Bc* [60]
		Egg laying amount	*Bc* [62]	
	Offspring	Survival rate	*Dm* [55,56] *Dc* [63]	
		Total number per brood	*Dm* [55] *Dc* [63]	*Dc* [63]
		Time of first brood	*Dm* [55] *Dc* [63]	*Dc* [63]
		Number of first brood	*Dm* [55] *Dc* [63]	*Dc* [63]
		Innate rate	*Dm* [55]	
		Mictic ratio increase	*Bc* [60,61]	*Bc* [60,61]
		Sex ratio	*Bc* [62]	
		Hatching rate abnormal	*Bc* [61]	*Bc* [61]
		Hatching percentage abnormal	*Bc* [61]	*Bc* [61]
	Hormone	Ecdysone receptor	*Dm* [48]	*Dm* [48]
Oxidative toxicity	Whole body	GST, CAT, SOD, GSH	*Dm* [46,48,54,56,58]	*Dm* [48]
Genetic toxicity	DNA damage	Comet tailing	*Dc* [63]	
Organ toxicity	Heart	Heart rate increase	*Dm* [46,54]	
Behavioral toxicity	Swimming	Activity (light)	*Dm* [57]	*Dm* [57]
		Activity (dark)	*Dm* [57]	
		Distance	*Dm* [57]	
		Speed	*Dm* [57]	
	Feeding	Ingestion rate	*Dm* [57]	
Neurology toxicity	Enzyme	AChE	*Dm* [46,48,54,56,58]	

*Dm, Daphnia magna; Dc, D. carinata; Bc, Brachionus calyciflorus.*

**Table 4:** Toxicity effects of commonly detected single PFASs on phytoplankton.

Classification	Damage/Effect	Impact Detail	PFOS	PFOA
Developmental toxicity	Growth	Chlorophyll content	*Ch* [45,74] *Cp* [75] *Sc* [45] *Mb* [76] *Mp* [76]	*So* [69] *Ps* [77] *Cp* [78] *Sc* [78] *Mb* [79] *Mp* [79]
		Carotenoid content	*Mb* [76] *Mp* [76]	*Mb* [79] *Mp* [79]
		Chloroplast structure	*Ch* [73]	
		Growth rate inhibition	*So* [71,72,73] *Ps* [80] *Ch* [45,81] *Cp* [75] *Sc* [45] *Mb* [76] *Mp* [76]	*Ps* [80] *Ch* [79] *Cp* [78] *Sc* [78] *Sm* [82] *Ga* [82] *Ds* [83] *Pt* [83] *Mb* [79] *Mp* [79]
Oxidative toxicity	Whole body	CAT, SOD, POD, BCA	*So* [72] *Ch* [73] *Cp* [75]	*So* [69] *Cp* [78] *Sc* [78]
	Lipid peroxidation	MDA	*Ch* [73]	*So* [69]
Cytotoxicity	Apoptosis	Cell membrane permeability	*So* [71] *Ch* [73]	*So* [71] *Cp* [78] *Sc* [78]
		Mitochondrial membrane potential	*So* [71]	*So* [71]

*So, Scenedesmus obliquus; Ps, Pseudokirchneriella subcapitata; Ch, Chlorella vulgaris; Cp, Chlorella pyrenoidosa; Sc, Selenastrum capricornutum; Sm, Skeletonema marinoi; Ga, Geitlerinema amphibium; Ds, Dunaliella salina; Pt, Phaeodactylum tricornutum; Mb, Myriophyllum sibiricum; Mp, Myriophyllum spicatum.*

## Data Availability

Not applicable.

## Supplementary Materials

The following supporting information can be downloaded at: https://www.mdpi.com/article/10.3390/ijerph192416729/s1, Table S1: Toxicity effects of other PFASs to aquatic invertebrates; Table S2: EC50, LC50, NOEC values of target PFASs and some substitutes to zooplankton; Table S3: Toxicity effects of other PFASs or substitutes to plankton; Table S4: EC50, LC50, NOEC values of target PFASs and some substitutes to phytoplankton.
