# Supplementary material for: Toxicity of Per- and Polyfluoroalkyl Substances to Aquatic Invertebrates, Planktons, and Microorganisms
Source: Int J Environ Res Public Health. 2022 Dec 13;19(24):16729. doi: 10.3390/ijerph192416729 (PMC9779086; doi:10.3390/ijerph192416729)
Supplement: Supplementary file 1 [file ijerph-19-16729-s001.zip › ijerph-2087406-supplementary.pdf]

International Journal of
Environmental Research and Public Health

Table S1. Toxicity effects of other PFASs on aquatic invertebrates.

**Classification**	**Damage/effect**	**Impact detail**	**PFNA**	**PFDA**	**PFBA**	**PFBS**
Oxidative toxicity	Whole soft tissues	CAT, SOD, AChE, GSH, GPX	*Pv* [21]	*Pv* [21]	Bss [38]	Bss [38]
	Lipid peroxidation	MDA	*Pv* [21]	*Pv* [21]		
Genetic toxicity	DNA damage	Strand break	*Pv* [21, 22]	*Pv* [21, 22]		
Overall toxicity	Enhanced integrated biomarker response		*Pv* [22]	*Pv* [22]		

Pv, *Perna viridis*; Bss, black shell shrimp; PFNA, perfluorononanoic acid; PFDA, perfluorodecanoic acid; PFBA, perfluorobutanoic acid; PFBS, perfluorobutane sulfonate.

Table S2. EC50, LC50, NOEC values of target PFASs and some substitutes on zooplankton.

Zooplankton	Pollutant	Effect	Indicator	Value (mg/L)	Refs
Daphnia magna	PFOS	LC50	24 h, 48 h lethal	193 (177–209), 63 (58–69)	[37]
	PFOA			298 (278–321), 181 (166–198)	
Isochrysis galbana	PFOS	LC50	72 h lethal	37.5	[41]
	PFOA			163.6	
Daphnia magna	PFOS	EC50	48 h activity inhibition	79.35	[46]
	PFOS	LC50	24 h lethal	150 (132.21-179.03)	[47, 49]
			48 h lethal	120.22 (99.32-145.01)	
			72 h lethal	70.65 (65.32-77.21)	
	PFOA		24 h lethal	366.12 (329.12-405.11)	
			48 h lethal	230.35 (210.21-243.11)	
			72 h lethal	102.41 (89.32-115.23)	
	PFOS	EC50	24 h activity inhibition	90.10 (82.32-99.11)	
			48 h activity inhibition	35.01 (32.34-39.03)	
			72 h activity inhibition	28.65 (24.12-36.90)	
	PFOA		24 h activity inhibition	238.21 (201.23-269.12)	
			48 h activity inhibition	120.12 (100.23-146.31)	
			72 h activity inhibition	97.21 (90.23-112.55)	
Daphnia magna	PFOS	LC50	48 h survival	130 (112-136)	[45]
			21 d adult survival	42.9 (31.7-56.4)	
		IC50	48 h immobility	67.2 (31.3-88.5)	
Daphnia pulicaria	PFOS	LC50	48 h survival	169 (136-213)	
			21 d adult survival	134 (103-175)	
Daphnia magna	PFOS	EC50	48 h mortality	21.16	[48]
	PFOA			120.91	
Daphnia magna	PFBSK	EC50	48 h immobility	> 100	[50]
		NOEC	21 d	571	
	PFOSK	EC50	48 h immobility	27-61	
			21 d	12	
			28 d	11	
		NOEC	21 d	12	
			28 d	7	

Daphnia magna	10:2 FTCA	LC50	20 d lethal	> 60	[51]
		EC50	Time to first brood	50 (46-54)	
			Reproductive day	48 (44-52)	
	10:2 FTuCA	LC50	20 d lethal	150 (120-200)	
		EC50	Time to first brood	287 (284-288)	
			Reproductive day	214 (199-221)	
Daphnia magna	$C_6HF_{11}O_3.H_3N$	EC50	48 h	> 102	[52]
		NOEC	21 d	4.17	
	AFPO	EC50	24 h immobility	599	[53]
			48 h immobility	480	
		NOEC	21 d	12.5	
	PFOS	EC50	48 h immobility	79.35	[54]
Daphnia magna	PFOS	LC50	24 h lethal	150.34 (132.21-179.03)	[55]
			72 h lethal	70.65 (65.32-77.21)	
	PFOS	EC50	48h lethal	103.16	[57]
	PFOA			248.4	
	PFOS	LC50	48h lethal	49.27	[58]
		EC50	48 h immobility	23.41	
	PFNA	LC50	48h lethal	80.93	
		EC50	48 h immobility	43.42	
	APFO	LC50	48h lethal	226.70	[59]
		EC50	48 h immobility	156.90	
Daphnia magna	PFBA	EC50	24 h inhibition	0.865 (0.858-0.871)	[65]
	5H 4:1 FTOH			1.332 (1.143-1.476)	
	PFOA			0.531 (0.506-0.555)	
	PFNA			0.481 (0.435-0.601)	
	PFDA			0.339 (0.310-0.366)	
	PFUnA			0.238	
	PFDoA			0.162 (0.152-0.174)	
	PFBA	EC50	48 h inhibition	0.848 (0.841–0.856)	
	5H 4:1 FTOH			1.222 (1.016–1.395)	
	PFOA			0.511 (0.446–0.617)	
	PFNA			0.326 (0.281–0.390)	
	PFDA			0.318 (0.278–0.345)	

	PFUnA			0.236 (0.163–0.327)	
	PFDoA			0.129 (0.098–0.160)	
Brachionus calyciflorus	PFOS	LC50	24 h lethal	61.8 mg/L	[60]
	PFOA			150.0 mg/L	
	TFA	LC50	24 h lethal	70 mg/L	[63]
	PFPrA			80 mg/L	
	PFBA			110 mg/L	
	PFPeA			130 mg/L	
	PFHxA			140 mg/L	
Chydorus sphaericus	PFBA	EC50	24 h inhibition	2.509 (2.409–2.607)	[65]
	5H 4:1 FTOH			1.393 (0.829–1.877)	
	PFOA			0.426 (0.223–0.537)	
	PFNA			0.121 (0.106–0.136)	
	PFDA			0.141 (0.110–0.173)	
	PFUnA			0.069 (0.056–0.082)	
	PFDoA			0.054 (0.044–0.086)	
	PFBA	EC50	48 h inhibition	2.160 (2.070–2.249)	
	5H 4:1 FTOH			0.842 (0.239–1.292)	
	PFOA			0.282 (0.122–0.345)	
	PFNA			0.060 (0.047–0.070)	
	PFDA			0.088 (0.051–0.124)	
	PFUnA			0.034 (0.022–0.042)	
	PFDoA			0.046 (0.034–0.081)	
Daphnia carinata	PFOS	LC50	24 h lethal	28.3 (19.5-38.1)	[66]
			48 h lethal	8.8 (6.4-11.6)	
		NOEC	24 h	10.0	
			48 h	5.0	
	PFOA	LC50	24 h lethal	103.3 (82.5-123.9)	
			48 h lethal	78.2 (54.9-104.9)	
		NOEC	24 h	25.0	
			48 h	15.0	
Daphnia pulex	PCS	LC50	48 h lethal	> 100	[68]
	Textile finishing agent			17.97 (13.67-20.85)	
	C4 finishing agent			64.61 (62.35-67.42)	

C6 finishing agent	85.58 (83.36-87.94)

PFBSK, potassium perfluorobutane sulfonate; PFOSK, potassium PFOS; FTCA, saturated fluorotelomer carboxylic acid; FTuCA, fluorotelomer unsaturated carboxylic acid; $C_6HF_{11}O_3.H_3N$, ammonium 2,3,3,3-tetrafluoro-2-(heptafluoropropoxy)-propanoate; APFO, ammonium perfluorooctanoate; 5H 4:1 FTOH, 2,2,3,3,4,4,5,5-octafluoro-1-pentanol; PFUnA, perfluoroundecanoic acid; PFDoA, perfluorododecanoic acid; PCS, perfluorinated butyl organic ammonium salt cationic surfactant.

Table S3. Toxicity effects of other PFASs or substitutes on plankton.

**Classification**	**Damage/effect**	**Impact detail**	**10:2 FTCA**	**10:2 FTuCA**	**PFPrA**	**PFBA**	**PFPeA**	**PFHxA**	**PFNA**	**F-53B**	**PFDoA**	**PFTeA**	**6:2 Cl-PFAES**	**GenX**	**FC-98**
Cumulative toxicity	Accumulation	Whole body								*So* [70]					
Developmental toxicity	Growth	Body size			*Bc* [63]	*Bc* [63]	*Bc* [63]								
		Chlorophyll content								*So* [70]					
		Growth rate inhibition			*Bc* [63]	*Bc* [63]	*Bc* [63]	*Bc* [63]			*So* [71]	*So* [71]	*Pl* [82]	*Pl* [82]	*Pl* [82]
Reproductive toxicity	Population	Growth inhibition							*Ds* [83] *Pt* [83]						
		Survival	*Dm* [51]	*Dm* [51]					*Dm* [58]						
		Total number per brood							*Dm* [58]						
		Time to first brood	*Dm* [51]	*Dm* [51]					*Dm* [58]						
		Number of first brood							*Dm* [58]						
		Brood day	*Dm* [51]	*Dm* [51]					*Dm* [58]						
		Egg size			*Bc* [63]	*Bc* [63]	*Bc* [63]	*Bc* [63]							
Oxidative toxicity	Whole body	SOD, CAT, ROS							*Dm* [58]	*So* [70]			*Pl* [82]	*Pl* [82]	*Pl* [82]
	Lipid peroxidation	MDA								*So* [70]					
Cytotoxicity	Apoptosis	CMP								*So* [70]	*So* [71]	*So* [71]			
		MMP								*So* [70]	*So* [71]	*So* [71]			
Behavioral toxicity	Feeding	Ingestion rate							*Dm* [58]						
	Enzyme	AChE							*Dm* [58]						

Bc, Brachionus calyciflorus; *Pl, Prorocentrum lima*.

PFPrA, perfluoropropionic acid; PFPeA perfluopentanoic acid; PFHxA, perfluorohexanoic acid; F-53B, chlorinated polyfluoroalkyl ether sulfonic acids; PFTeA, perfluorotetradecanoic acid; 6:2 Cl-PFAES, 6:2 chlorinated polyfluoroalkyl ether sulfonic acids; GenX, [2,3,3,3-tetrafluoro-2-(1,1,2,2,3,3,3-heptafluoropropoxy)-propanoic acid; FC-98, perfluoroethylcyclohexane sulfonate; CMP, cell membrane permeability; MMP, mitochondrial membrane potential.

Refer other denotes to Table S1, S2.

Table S4. EC50, LC50, NOEC values of target PFASs and some substitutes on phytoplankton.

Phytoplankton	Pollutant	Effect	Indicator	Value (mg/L)	Refs
Scenedesmus obliquus	PFOA	EC50	96 h inhibition	139.23	[69]
	Cl-PFESA	EC50	72 h inhibition	13.4	[70]
	PFOS		72 h fluorescence inhibition	78.02 (75.52–80.02)	[71]
	PFDoA		72 h fluorescence inhibition	160.28 (155.37–165.19)	
			72 h cell density inhibition	112.38 (108.7–116.06)	
	PFTeA		72 h fluorescence inhibition	71.24 (69–73.10)	
			72 h cell density inhibition	49.72 (47.12–51.95)	
	PFOS	EC50	96 h cell density inhibition	75	[72]
			96 h absorbance inhibition	98	
			96 h chlorophyll (a) inhibition	95	
Pseudokirchneriella subcapitata	PFOS	EC50	72 h growth inhibition	35.0 (34.2-35.5)	[74]
	PFOA			96.2 (88.6-113.7)	
	PF-656			43.0 (41.1-44.9)	
	$C_6HF_{11}O_3.H_3N$	EC50	72 h growth inhibition	> 107	[52]
	PFBA	EC50	120 h photosynthesis inhibition	1.225 (1.00–1.50)	[75]
	5H 4:1 FTOH			4.853 (4.10–5.80)	
	PFOA			1.807 (1.76–1.86)	
	PFNA			1.038 (0.98–1.10)	
	PFDA			0.851 (0.64–1.12)	
	PFUnA			0.565	
	PFDoA			0.394	
	PFOS		72 h growth inhibition	35.0 (34.2–35.5)	[76]
Chlorella vulgaris	PFOS	EC50	96 h cell density inhibition	81.6 (69.6-98.6)	[45]
			96 h chlorophyll (a) inhibition	88.1 (71.2-104)	
		NOEC	96 h cell density inhibition	8.2 (6.4-13.0)	
			96 h chlorophyll (a) inhibition	9.6 (7.6-16.5)	
	OBS fluorine protein	EC50	48 h growth inhibition	1.228	[77]
	foam extinguishing		72 h growth inhibition	1.101	
	agent		96 h growth inhibition	0.909	
	PFHxA	EC50	72 h growth inhibition	4032.44 ± 200.99	[78]
	PFHpA			1896.75 ± 94.66	

	PFOA			977.21 ± 49.69	
	PFNA			496.57 ± 23.20	
Chlorella pyrenoidosa	PFOS	EC50	96 h growth inhibition	320	[80]
	PFOA	EC50		190.99 (184.97-197.01)	[81]
Selenastrum capricornutum	PFOA	EC50		207.46 (182.61-232.31)	
	PFOS	EC50	96 h cell density inhibition	48.2 (45.2-51.1)	[45]
			96 h chlorophyll (a) inhibition	59.2 (50.9-67.4)	
		NOEC	96 h cell density inhibition	5.3 (4.6-6.8)	
			96 h chlorophyll (a) inhibition	16.6 (8.5-28.1)	
Isochrysis galbana	PFOS	EC50	72 h growth inhibition	37.5 (31.1–45.2)	[41]
	PFOA			163.6 (131.7–203.2)	
	PFOS	NOEC		7.5	
	PFOA			25	
Skeletonema marinoi	PFHxA	EC50	72 h growth inhibition	1482.33 ± 72.23	[78]
	PFHpA			873.74 ± 43.69	
	PFOA			368.5 ± 16.56	
	PFNA			194.91 ± 9.28	
Geitlerinema amphibium	PFHxA			998.69 ± 50.25	
	PFHpA			516.97 ± 25.48	
	PFOA			248.44 ± 12.42	
	PFNA			129.94 ± 4.64	
Dunaliella salina	PFOA	EC50	96 h growth inhibition	668.671	[83]
	PFNA			156.585	
Phaeodactylum tricornutum	PFOA			351.775	
	PFNA			65.127	
Myriophyllum sibiricum	PFOA	EC50	21 d plant length inhibition	50.0 (19.4-80.6)	[85]
			21 d root number inhibition	44.6 (30.1-59.3)	
			21 d root length inhibition	42.9 (26.0-58.2)	
			21 d longest root inhibition	43.3 (18.4-68.3)	
			21 d node number inhibition	55.2 (0-144.6)	
			21 d wet mass inhibition	70.4 (36.8-104.1)	
			21 d dry mass inhibition	39.6 (24.9-54.4)	
			21 d Chlorophyll (a) inhibition	86.2 (38.7-133.7)	
			21 d Chlorophyll (b) inhibition	99.9 (8.8-191.0)	

			21 d carotenoids inhibition	135.1 (29.2-241.1)	
Myriophyllum spicatum			21 d plant length inhibition	31.8 (9.9-53.8)	
			21 d root number inhibition	80.5 (29.1-131.9)	
			21 d root length inhibition	56.9 (22.1-91.8)	
			21 d longest root inhibition	69.3 (42.5-96.2)	
			21 d node number inhibition	44.8 (23.7-65.9)	
			21 d wet mass inhibition	37.3 (10.7-63.9)	
			21 d dry mass inhibition	40.2 (10.2-70.1)	
			35 d Chlorophyll (a) inhibition	110.4 (38.9-181.8)	
			35 d Chlorophyll (b) inhibition	117.9 (0-308.4)	
			35 d carotenoids inhibition	294.2 (0-707.3)	
Myriophyllum sibiricum	PFOS	EC50	28 d plant length inhibition	9.8 (0-66.1)	[84]
			28 d root number inhibition	6.8 (5.0-8.5)	
			28 d root length inhibition	14.1 (9.9- 18.4)	
			28 d longest root inhibition	10.2 (5.2-15.1)	
			28 d node number inhibition	11.9 (3.4-20.3)	
			28 d wet mass inhibition	4.6 (3.0-6.2)	
			28 d dry mass inhibition	13.7 (9.2-18.3)	
			28 d Chlorophyll (a) inhibition	18.8 (10.1-27.5)	
			28 d Chlorophyll (b) inhibition	22.2(11.1-33.4)	
			28 d carotenoids inhibition	28.5 (21.5-35.5)	
Myriophyllum spicatum			28 d plant length inhibition	22.7 (13.6-31.9)	
			28 d root number inhibition	23.6 (15.4-31.8)	
			28 d root length inhibition	17.3 (9.3-25.3)	
			28 d longest root inhibition	22.8 (16.3-29.3)	
			28 d node number inhibition	20.6 (6.4-34.8)	
			28 d wet mass inhibition	17.8 (13.9-21.7)	
			28 d dry mass inhibition	16.6 (11.6-21.6)	
			28 d Chlorophyll (a) inhibition	30.7 (22.4-39.1)	
			28 d Chlorophyll (b) inhibition	33.3 (22.2-44.4)	
			28 d carotenoids inhibition	39.4 (27.3-51.5)	
Scenedesmus quadricanda	Textile finishing agent	EC50	72 h lethal	88.32 (52.12-171.50)	[68]
Paramecium caudatum	PFOS		Swam backward inhibition	14.90 ± 2.05	[87]
	PFOA			175.61 ± 51.34	

	PFNA			45.8 ± 9.33	
	PFDA			31.05 ± 5.19	
Lemna gibba	PFOS	EC50	96 h frond number inhibition	59.1 (51.5-60.3)	[45]
			96 h wet weight inhibition	31.1 (22.2-36.1)	

Cl-PFESA, chlorinated polyfluorinated ether sulfonate; PF-656, fluorinated and hydroxylated polyether; OBS, sodium p-perfluorous nonenoxybenzenesulfonate; PFHpA, perfluoroheptanoic acid.
